# Establishment of a Chikungunya virus pseudotype system strictly dependent on viral protein expression

**DOI:** 10.1099/jgv.0.002138

**Published:** 2025-08-11

**Authors:** Atsushi Tanaka, Takayuki Miyazawa

**Affiliations:** 1 Division of Research Animal Laboratory and Translational Medicine, Research and Development Center, Osaka Medical and Pharmaceutical University, Takatsuki, Osaka 569-8686, Japan; 2 Laboratory of Virus-Host Coevolution, Institute for Life and Medical Sciences, Kyoto University, Sakyo-ku, Kyoto 606-8507, Japan; 3 Kyoto Animal Human Organism Research Institute, Nakagyo-ku, Kyoto, Japan

**Keywords:** Chikungunya virus, pseudotype virus, SARS-CoV-2

## Abstract

Chikungunya virus (CHIKV) is an enveloped RNA virus that causes Chikungunya fever in humans. It is classified as an arbovirus (arthropod-borne virus) and is transmitted by mosquitoes. Therefore, CHIKV can replicate in many types of cells derived from mammals or insects. In this study, we tried to establish the widely useable CHIKV pseudotype system, adapting various viral species, and we demonstrated the production of Chikungunya pseudotype virus bearing the envelope protein from two different viral families, *Coronaviridae* or *Rhabdoviridae*, i.e. severe acute respiratory syndrome coronavirus 2 spike protein or vesicular stomatitis virus glycoprotein, respectively. We found that the capsid protein of CHIKV is not always necessary in the formation of CHIKV-based pseudotypes, but that the capsid protein increases the efficiency of expression of the sub-genomic RNA which codes the labelled genes. Our established pseudotype virus-producing system supplied a sufficient titre of virions for application to most virological experiments that showed more than 10^4^ focus-forming units per millilitre. The pseudotype infections were strictly dependent on compatibility between the viral envelope protein and its receptor, and there was no false-positive background infection. Our established pseudotype virus system can be used as a robust platform to study various virus infections and for screening and in-depth evaluation of neutralizing antibodies and antiviral agents.

## Introduction

Chikungunya virus (CHIKV) is a mosquito-borne alphavirus in the family *Togaviridae*. It is the causative agent of Chikungunya fever, which occurs mainly in tropical regions such as those in Africa, South Asia and Southeast Asia [1]. Chikungunya fever usually begins 2–12 days after the mosquito bite. It is characterized by the sudden onset of a high fever that is frequently accompanied by severe joint pain, muscle pain, headache, nausea, fatigue and rash, lasting for several days, and this acute phase is often followed by a chronic phase, characterized by persistent and crippling arthralgia [2,4]. During CHIKV infection, viraemia lasts 5–7 days, and high viral loads of up to 10^9^ viral RNA copies per millilitre of blood plasma can be detected in the early stages of infection [5,6].

CHIKV has a single positive-stranded RNA genome of 11.8 kbp encoding four nonstructural and five structural proteins. The structural proteins are translated from a subgenomic RNA as a single polyprotein, which is processed co-translationally into five structural proteins: capsid, E3, E2, 6K and E1 [7]. These structural proteins form two T=4 quasi-icosahedral symmetry layers: the viral surface lipid membrane with a dia. of 65–70 nm containing 80 viral envelope spikes that consist of 240 copies of the E1–E2 heterodimer, and the icosahedral nucleocapsid core comprised of 240 copies of the capsid [8,11]. The viral envelope E2 glycoprotein is responsible for the binding to the cell surface receptor, and the E1 protein serves as a fusion protein [9,12,17].

During assembly of CHIKV virus particles, the CHIKV genomic RNA is selectively packaged into the capsid core through interaction between a specific packaging signal in the nsP2 region of the viral genomic RNA and the capsid core [18]. The viral capsid core and envelope proteins are arranged in organized lattices linked via the interaction of the E2 cytoplasmic tail/endodomain with the capsid protein [19]. Such interactions between viral components allow for the effective formation of viral particles [19,20]. The nucleocapsid core of the alphavirus is assembled not only in the infected cells in the absence of envelope protein expression [21,22] but also *in vitro* when incubated with RNA [23]. It has been reported that the lattice formed of viral envelope glycoprotein spikes can itself promote nucleocapsid formation [24,27], and the cytoplasmic nucleocapsids undergo rearrangements during budding [21,24, 28], suggesting that interactions between the two lattices may influence the mature particle structure.

Although it has been reported that the interaction between the viral genomic RNA and the capsid protein and the interaction between the capsid protein and the envelope E2 protein are important for the formation of infectious virus particles, as described above, Zhang *et al*. recently constructed an infectious CHIKV with complete capsid deletion as a live attenuated vaccine candidate [29,30] was indicating that these interactions were not necessarily required for viral particle formation [29,30].

CHIKV has been shown to infect various types of vertebrates as potential natural animal hosts, as well as a wide range of cell lines *in vitro* [31,32], and to target a wide range of organs and tissues, including the joints, skin, liver, muscle and secondary lymphoid organs [32,33]. The broad range of cell tropism that CHIKV can infect is likely due to the ubiquitous expression of putative receptors to which CHIKV envelope glycoproteins bind across a range of species and cell types [34,36], as well as a CHIKV replication system that can function efficiently across a range of species and cell types [37].

To date, pseudotype viruses bearing envelope proteins of different virus species on the surface of virus particles have been constructed using recombinant virus vectors with marker genes incorporated into the genome, such as lentivirus vector, mouse retrovirus vector and vesicular stomatitis virus (VSV) vector, and pseudoviruses expressing CHIKV surface envelope proteins (E3, E2 and E1) have also been constructed using these virus vectors [38,41]. On the other hand, although there are abundant replicon/helper systems for alphaviruses [37,42,44], there are no systems producing the pseudotype virus bearing an alternative envelope protein that is not of alphavirus origin.

In this study, we tried to construct CHIKV-based pseudotyped viruses to establish a pseudotype virus system that produces virus in high titre, can be used in a wide range of target cells and can be prepared conveniently. Our constructed pseudotyped virus bears an envelope protein that is from a viral family distinct from alphaviruses such as the severe acute respiratory syndrome coronavirus 2 spike protein (CoV-2-S) or VSV glycoprotein (VSV-G), and its genome has the labelled gene, which is substituted into the structural protein coding region and expressed as subgenomic RNA. This pseudotype system has the potential to serve as a robust platform for the study of various virus infections and for screening the corresponding neutralizing antibodies and antiviral agents.

## Methods

### Cells

A baby hamster kidney fibroblast cell line (BHK) and its derivative [BHK/hACE2 (human angiotensin-converting enzyme 2)], an African green monkey kidney cell line (Vero), a human embryonic kidney cell line (293T) and a human lung cancer cell line (Calu3) were maintained in Dulbecco’s modified minimum essential medium supplemented with 10% FBS, 50 units per millilitre penicillin and 50 µg ml^−1^ streptomycin. To generate BHK cells stably expressing hACE2 (GenBank accession no. NM_001371415), which we designated BHK/hACE2, an hACE2 ORF sequence was amplified by reverse transcription-PCR from 293T first-strand cDNA synthesized using a Verso cDNA Synthesis Kit (Thermo Scientific, Rockford, IL) with a random hexamer. The sequences of the PCR primers for the hACE2 ORF were hACE2/sense (5′-tttctcgagacg**ATG**TCAAGCTCTTCCTGGCTCCTTC-3′) and hACE2/antisense (5′-aaagcggccg**C****TA**AAAGGAGGTCTGAACATCATC-3′). Their respective restriction enzyme sites, *Xho* I and *Not* I, are underlined, and the positions of initiation and termination codons are shown in bold font. The hACE2 PCR products were cloned into the retroviral expression vector plasmid pLPCX, a retroviral vector containing a gene encoding resistance to puromycin. VSV-G-pseudotyped murine retrovirus carrying the human ACE2 gene was generated by co-transfecting 293T cells with the pLPCX-hACE2, pMD-gag-pol [45], and a VSV-G-expressing plasmid (Clontech, Palo Alto, CA), pVSV-G [46]. A murine retrovirus packaging vector, pLPCX-hACE2 (VSV-G), was used to transduce BHK-21 cells in the presence of 4 µg ml^−1^ polybrene. The transduced BHK-21 cells were selected by culture in a medium containing puromycin at 1 µg ml^−1^ for over 2 weeks. The hACE2 expression of BHK/hACE2 cells was confirmed by immunostaining using a VECTASTAIN ABC kit (Vector Laboratories, Burlingame, CA) with anti-ACE2 rabbit polyclonal antibody (21115-1-AP) (Proteintech Group, Rosemont, IL), as shown in Fig. S1, available in the online Supplementary Material.

### Plasmids and antibodies

**Fig. 1.**
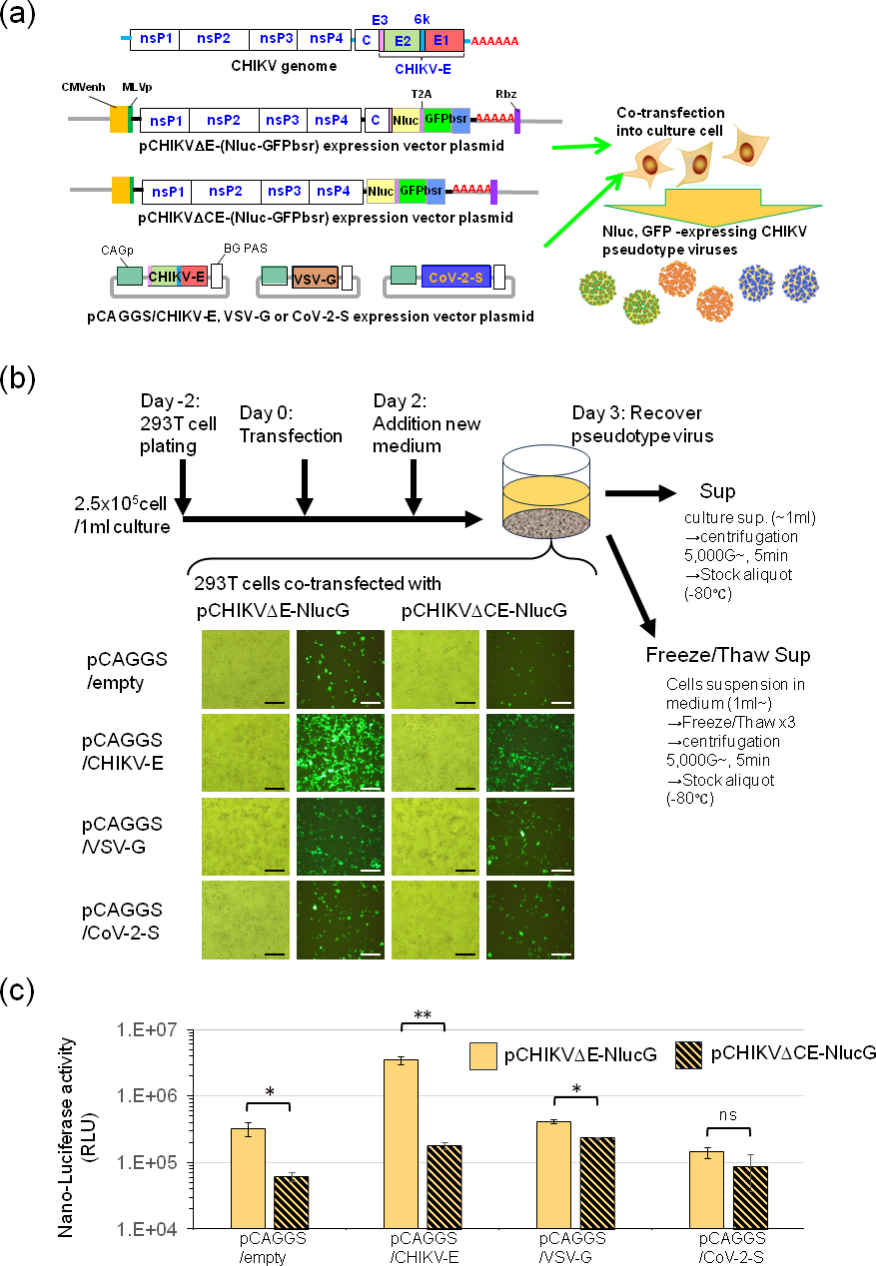
CHIKVΔE-NlucG/viral envelope protein pseudotype design and formulation. (**a**) Shown are the rCHIKV-gRNA expression vector plasmids, pCHIKVΔE-NlucGFPbsr and pCHIKVΔE-luc, and the viral envelope expression vectors, pCAGGS/CHIKV-E, pCAGGS/VSV-G and pCAGGS/CoV-2-S. These expression vector plasmids were constructed as described in the Methods section.Abbreviations: nsP1-4: the nonstructural protein genes of CHIKV strain Ross. C and E: the capsid (C) gene and envelope (E) gene (E3, E2, 6K and E1) (i.e. the structural protein genes) of CHIKV strain Ross. CMVenh: the human cytomegalovirus (CMV) early enhancer sequence. MLVp: the murine leukaemia virus promoter sequence. Nluc: Nano luciferase gene. luc: firefly luciferase gene. T2A: *Thosea asigna* virus 2A sequence. GFPbsr: CpG-free GFP::Bsr fusion gene (Invitrogen). HDV: hepatitis D virus. Rbz: ribozyme sequence. AAAAA: polyA sequence. CAGp: the CMV early enhancer/chicken β-actin promoter of the pCAGGS expression vector. CHIKV-E, CHIKV envelope gene. VSV-G, vesicular stomatitis virus glycoprotein gene. CoV-2-S, SARS-CoV-2 spike protein gene. BG PAS, β-globin poly(A) signal. (**b**) Time course of the preparation of pseudotype samples. 293T cells were plated at 2.5×10^5^ cells/1 ml culture 1 or 2 days before transfection (day 0). At day 1, 293T cells were co-transfected with pCHIKVΔE (or ΔCE)-NlucGFPbsr expression vector plasmid and pCAGGS/empty, pCAGGS/CHIKV-E, pCAGGS/VSV-G or pCAGGS/CoV-2-S vector plasmid, and then, the GFP expression levels in these transfected cells were detected by fluorescence microscopy at 3 days after transfection, and then, the pseudotype virus samples were harvested as the culture supernatants (Sup) and the freeze/thaw-treated cell suspension with culture supernatant (F/T sup) as described in the Methods section. A scale bar indicates 200 µm. (**c**) Detection of NanoLuc luciferase (NLuc) activity expressed in 293T cells co-transfected with pCHIKVΔE-NlucGFPbsr and pCAGGS/(empty, CHIKV-E, VSV-G or CoV-2-S) vector. The NLuc activity expressed in relative light units (RLU) of cell lysate equivalent to 1 in 500 of the volume of cell lysate in one well of a 96-well multiplate was measured, and the results shown are representative of three independent experiments and are expressed as the mean±sd. Statistical significance was evaluated by an unpaired two-tailed *t*-test. *Significant at *P*<0.05; **significant at *P*<0.01; ns: not significant.

Genomic RNA of the CHIKV Ross strain was extracted by using a QIAamp viral RNA minikit (Qiagen, Hilden, Germany), and the first-strand cDNA was synthesized using a SuperScript III reverse transcriptase kit (Invitrogen Carlsbad, CA) with a random hexamer and/or a poly(dT)20NotIXbaI primer (5′-AAATCTAGAGCGGCCGCTTTTTTTTTTTTTTTTTTTT-3′) [39]. The CHIKV Ross strain genome sequence (GenBank accession no. AF490259) was introduced into the RNA synthesis start site of the pCXbsr retrovirus vector, where the region downstream of the CMV enhancer-MuLV promoter region and its structural protein region in which the envelope protein region (E) or capsid and envelope protein (CE) of this CHIKV genome was substituted with the construct NanoLuc luciferase (NLuc) gene-T2A sequence-CpG-free GFP::Bsr fusion gene (Invitrogen), designated NlucGFPbsr. To produce the viral RNA effectively, a hepatitis D virus ribozyme sequence (Rbz) [47] was introduced into the end of the poly A region of the CHIKV genome. The structure of these tagged defective CHIKV genome (recombinant CHIKV genome RNA: rCHIKV-gRNA) expression plasmids, named pCHIKVΔE-NlucGFPbsr and pCHIKVΔCE-NlucGFPbsr, is shown in Fig. 1.

The CHIKV-E glycoprotein expression plasmids, pCAGGS/CHIKV-E Ross strain, were described previously [39,48]. The VSV-G protein expression plasmid, pCAGGS/VSVG, was described previously [49]. The SARS-CoV-2 Wuhan strain spike protein expression plasmids, pCAGGS/CoV-2-S, encoding the codon-optimized sequence of the SARS-CoV-2 spike protein, were kindly provided by Y. Matsuura (Osaka University, Osaka, Japan) [50]. The SARS-CoV-2 Omicron BA.1 spike protein expression plasmid, pCAGGS/CoV-2-BA1-S, which encodes the codon-optimized sequence of the SARS-CoV-2 Omicron BA.1 spike protein (GenBank accession no. PV871806), was synthesized by using the pCAGGS/CoV-2-S as a base and coding the amino acid changes, deletions and insertions that are different from the amino acid sequences of SARS-CoV-2 Wuhan strain spike protein as follows: A67V, H69-, V70-, T95I, G142D, V143-, Y144-, Y145-, N211I, L212-, 215 (insertion: EPE), G339D, R346K, S371L, S373P, S375F, K417N, N440K, G446S, S477N, T478K, E484A, Q493R, G496S, Q498R, N501Y, Y505H, T547K, D614G, H655Y, N679K, P681H, N764K, D796Y, N856K, Q954H, N969K and L981F, that showed in the website: Covariant (https://covariants.org/).

The antibodies used here were as follows: anti-VSV-glycoprotein mouse monoclonal antibody, clone 8G5F11 (catalogue no. MABF2337-100UG; Merck, USA); anti-SARS-CoV-2 spike protein recombinant rabbit monoclonal antibody, clone HL257 (catalogue no. MA5-36253; Thermo Fisher Scientific, Waltham, MA); anti-SARS-CoV-2 (2019-nCoV) spike neutralizing antibody, mouse monoclonal antibody, clone #43 (catalogue no. 40591-MM43; Sino Biological, Beijing, China); and anti-ACE2 rabbit polyclonal antibody (catalogue no. 21115–1-AP; Proteintech Group).

### CHIKV-based pseudotypes

The rCHIKV-gRNA expression vector plasmid, pCHIKVΔE-NlucGFPbsr or pCHIKVΔCE-NlucGFPbsr, was co-transfected with the viral envelope expression vector, pCAGGS/CHIKV-E, pCAGGS/VSV-G, pCAGGS/CoV-2-S or pCAGGS/CoV-2-BA1-S, to subconfluent 293T cells that were plated 2 days before transfection. Culture supernatants and the cell suspension of cells freeze/thaw-treated three times with culture supernatants were harvested after 3 days of incubation at 37 °C in a CO_2_ incubator and clarified by low-speed (~5,000 ***g***, 5 min) centrifugation. Then the aliquots were stored at −80 °C until use (Fig. 1b). The serially diluted 50 µl supernatants, including pseudotypes, were inoculated into the cells in the wells of a 96-well multiplate seeded with subconfluent to confluent cells. Their infectivities were determined by counting GFP-positive cell foci consisting of one to two cells, or by measuring the NLuc activities with a Nano-Glo^®^ Luciferase Assay System (Promega, Madison, WI) at 1 day after inoculation.

### CHIKV-based pseudotype neutralization assay

The diluted samples including ~10^2^ focus-forming units (FFU) of pseudotypes were incubated with serially diluted antibodies at 37 °C for 30 min and then inoculated into the target cells in wells of a 96-well multiplate seeded with subconfluent to confluent cells. After 1 day of incubation, pseudotype titres were determined by counting GFP-positive cell foci.

## Results and discussion

In this study, we described the establishment of a CHIKV pseudotype virus-producing system that can be applied to produce various types of envelope viruses. The indicator gene in the rCHIKV-gRNA expression vector plasmid was expressed not only in human cells but also in various types of cells from mammals and insects of different phyla [51].

**Fig. 2.**
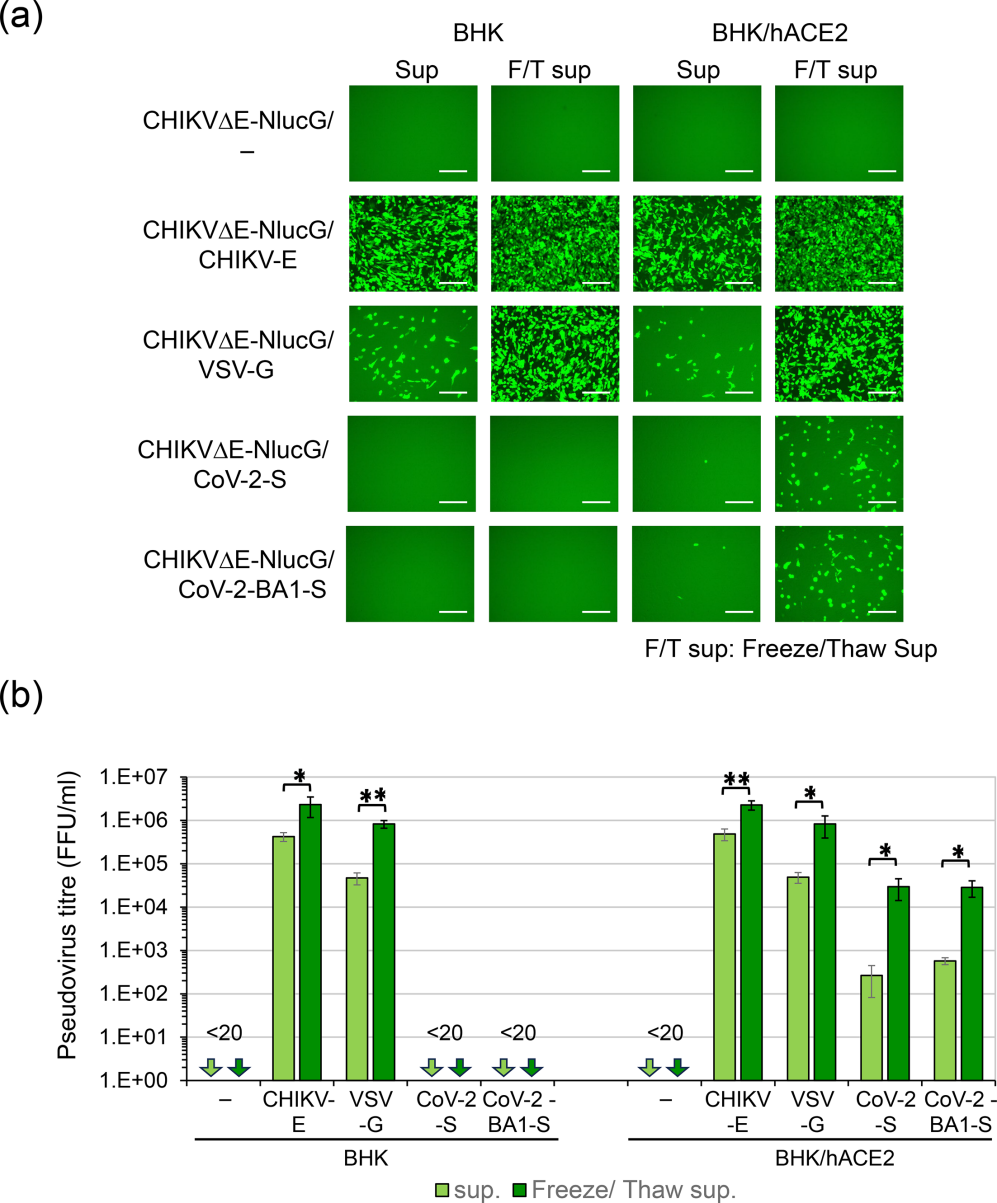
Infectivity of CHIKVΔE-NlucG/viral envelope protein pseudotypes. (**a**) Non-diluted CHIKVΔE-NlucG/viral envelope protein pseudotype samples (50 µl) obtained as Sup and F/T sup were inoculated into BHK and BHK-hACE2 cell culture in 96-well multiplate, and GFP-expressing cells detected by fluorescence microscopy at 1 day after inoculation are shown. A white scale bar indicates 100 µm. (**b**) The titre of CHIKVΔE-NlucG/viral envelope protein pseudotype samples was determined by counting GFP-positive cell foci as described in the Method section. Data shown are representative of three independent experiments and are expressed as the mean±sd. Statistical significance was evaluated by an unpaired two-tailed *t*-test. *Significant at *P*<0.05; **significant at *P*<0.01.

In previous studies, viral samples of native CHIKV have been collected from the culture supernatant of virus-infected BHK cells, a highly productive cell line of CHIKV. CHIKV pseudotypes (murine retrovirus-based and VSV-based) bearing the CHIKV membrane envelope protein, CHIKV-E, have been collected from the culture supernatant of 293T cells transfected with pseudotype-producing gene expression plasmids [39,40, 48]. In this study, we initially tried to prepare the pseudotypes by collecting the culture supernatants of cells co-transfected with a rCHIKV-gRNA expression vector plasmid, pCHIKVΔE-NlucGFPbsr or pCHIKVΔCE-NlucGFPbsr, and viral envelope expression vector plasmids (Fig. 1a). 293T cells transfected with pCHIKVΔE-NlucGFPbsr (or pCHIKVΔCE-NlucGFPbsr) expressed NLuc and GFP (Fig. 1b, c), and the numbers of GFP-expressing cells were increased in the cells co-transfected with the CHIKV-E or VSV-G protein expression plasmid but not in those transfected with the empty or SARS-CoV-2-S protein expression plasmid, by infecting the surrounding cells with the produced pseudotypes. And also, an increase of NanoLuc luciferase activity was observed in the cells co-transfected with pCHIKVΔE-NlucGFPbsr and pCAGGS/CHIKV-E, with pCHIKVΔCE-NlucGFPbsr and pCAGGS/CHIKV-E or with pCHIKVΔCE-NlucGFPbsr and pCAGGS/VSV-G (Fig. 1c). Then, we obtained the native-form CHIKV pseudotype bearing the CHIKV-E, which we designated CHIKVΔE-NlucG/CHIKV-E, and the pseudotype bearing the VSV-G, which we named CHIKVΔE-NlucG/VSV-G, from the culture supernatants of 293T cells transfected with each, in titres of more than 10^5^ FFU per millilitre and more than 10^4^ FFU per millilitre, respectively, in inoculated BHK cells (Fig. 2a).

On the other hand, the BHK cells expressed no human ACE2. They were completely resistant to SARS-CoV-2 infection (Fig. S2), and CHIKV pseudotype bearing the SARS-CoV-2-S proteins named CHIKVΔE-NlucG/CoV-2-S and CoV-2-BA1-S also could not infect BHK cells (Fig. 2a). Therefore, we established human ACE2-expressing BHK cells, designated BHK/hACE2 cells, that were highly susceptible to native SARS-CoV-2 (Fig. S2) and then inoculated them with CHIKVΔE-NlucG/CoV-2-S (or BA1-S). Although we detected the pseudotypes bearing the SARS-CoV-2-S or BA1-S, their titres were only ~10^2^ FFU per millilitre (Fig. 2a).

Previous studies reported that the viral core of alphaviruses containing the viral genomic RNA was enveloped with the cell surface plasma membrane, which includes the viral envelope protein. They budded from the cell surface [52,53]. At the same time, coronavirus genomic RNA complexed with nucleocapsid proteins was enveloped at the endoplasmic reticulum–Golgi interface, and viral particles were budded into the lumen of the intermediate compartment [54,56]. Recently, Reis *et al*. showed that some CHIKV particles acquire an envelope intracellularly without budding from the cell surface membrane [57]. We therefore suspected that a large proportion of CHIKVΔE-NlucG/CoV-2-S accumulated in the cytoplasmic compartment. We then used freeze and thaw treatments of cells to break the plasma membranes and release the CHIKVΔE-NlucG/CoV-2-S pseudotype virion from the cytoplasmic compartments. As expected, we were able to recover the infectious CHIKVΔE-NlucG/CoV-2-S pseudotypes from the culture supernatant of cells with plasma membranes fractured by freeze and thaw treatments at levels 100 times higher than the corresponding levels from the culture supernatant of living cells, and we found that the CHIKVΔE-NlucG/CoV-2-S and /CoV-2-BA1-S pseudotypes infect BHK/hACE2 cells, but not BHK cells (Fig. 2). This freeze and thaw treatment of cells increased the recovery of the titres of CHIKVΔE-NlucG/CHIKV-E and CHIKVΔE-NlucG/VSV-G pseudotypes by 5- and 17-fold compared to the titres in the culture supernatants of living cells. The pseudotypes bearing no viral envelope protein, CHIKVΔE-NlucG/- (empty), had no infectivity (Fig. 2).

**Fig. 3.**
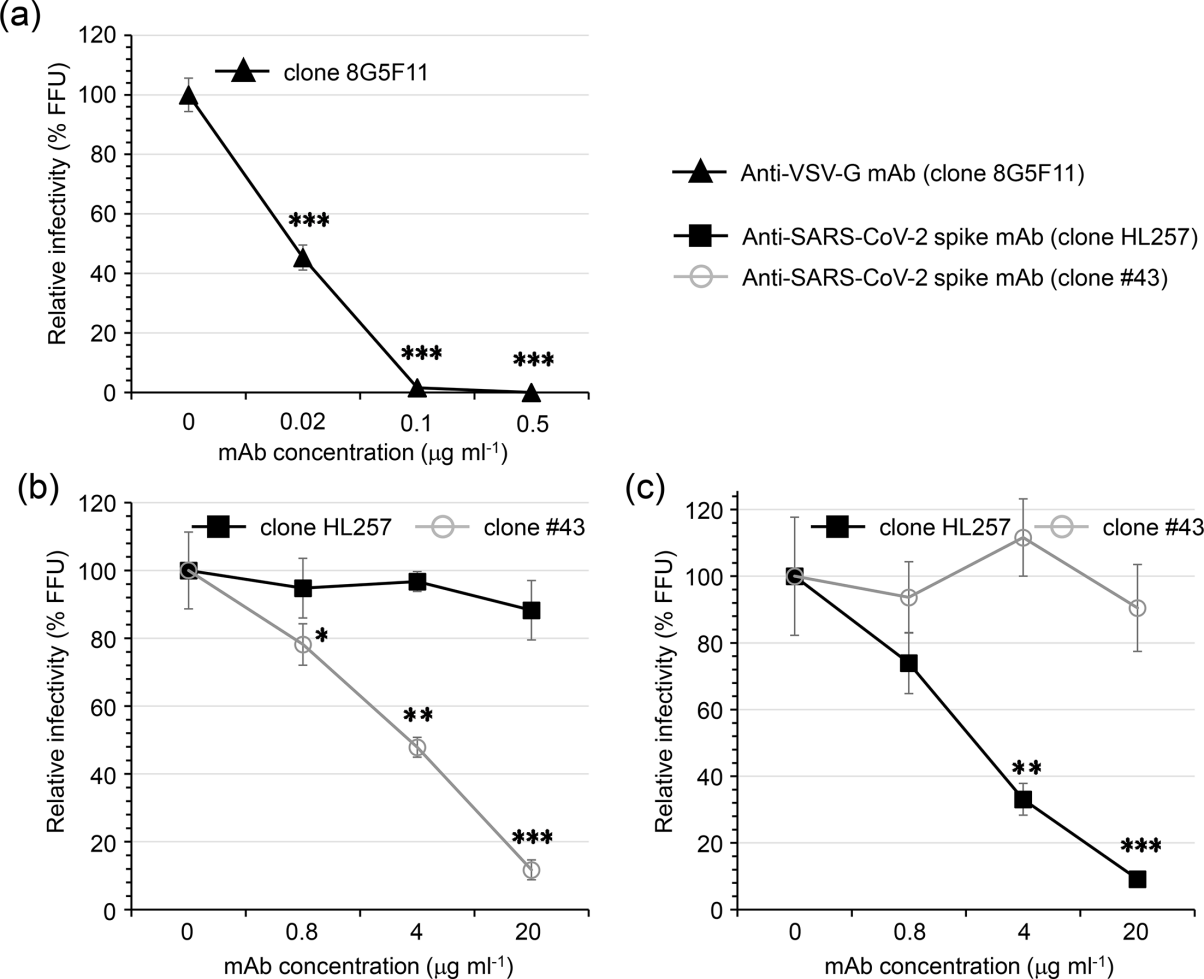
Neutralizing activity of anti-viral envelope protein detected by using the CHIKVΔE-NlucG/viral envelope pseudotype. Neutralizing activity of anti-VSV-glycoprotein mAb (clone 8G5F11) and anti-SARS-CoV-2 spike protein mAbs (clone HL257 and clone #43) (see the Methods section) detected by neutralization assay using the (**a**) CHIKVΔE-NlucG/VSV-G, (**b**) CHIKVΔE-NlucG/CoV-2-S and (**c**) CHIKVΔE-NlucG/CoV-2-BA1-S, respectively. The plots shown are representative of three independent experiments performed in duplicate or triplicate. Data shown are expressed as the mean±sd. Statistical significance was evaluated by an unpaired two-tailed *t*-test. *Significant at *P*<0.05; **significant at *P*<0.01; ***significant at *P*<0.001.

The CHIKVΔE-NlucG/VSV-G and CHIKVΔE-NlucG/CoV-2-S (or BA1-S) pseudotype infectivities were neutralized with specific anti-envelope antibodies against each envelope protein (Fig. 3), suggesting that the infections of CHIKV pseudotypes were strictly dependent on compatibility between the viral envelope protein and its receptor, and there was no false-positive background infection.

In this study, we found that the anti-SARS-CoV-2 spike protein recombinant rabbit monoclonal antibody clone HL257 recognizes both the spike antigen of prototype CoV-2 and Omicron BA.1 (Fig. S3) but neutralizes only CHIKVΔE-NlucG/CoV-2-BA1-S (Fig. 3). Therefore, SARS-CoV-2-BA.1 escaped neutralization of HL257 by changing the secondary or tertiary structure of its receptor-binding domain (RBD).

In the production of pseudotypes bearing the CHIKV-E or VSV-G, the pseudotypes infect surrounding cells transfected with envelope protein expression vectors, further increasing the number of producing cells bearing the CHIKVΔE-NlucG or CHIKVΔCE-NlucG genome. On the other hand, in the production of CHIKVΔE-NlucG/CoV-2-S (or BA1-S) pseudotypes, the produced pseudotypes cannot spread the infection to surrounding cells because there is almost no ACE2 expression in 293T cells. Thus, pseudotype-producing cells did not increase (Fig. 1b). This may be one of the reasons that the titres of CHIKVΔE-NlucG/CoV-2-S (or BA1-S) pseudotypes were lowest among the pseudotypes prepared in this study. Therefore, we tried to prepare the CHIKVΔE (orΔCE)-NlucG/CoV-2-S (or BA1-S) pseudotype by using ACE2-expressing cells, but the cells transfected with the viral envelope expression vector, pCAGGS/CoV-2-S or pCAGGS/CoV-2-BA1-S, formed large fusion cells in poor condition, so the pseudotypes could not be generated (Fig. S4).

Of course, in order to produce high-titre pseudotypes, it is crucial to produce a large number of cells expressing the rCHIKV-gRNA. Therefore, it is thought that pseudotype production can be efficiently increased by using transfection reagents that can efficiently introduce vector DNA into target cells or by using cell lines that show higher transfection efficiency. It is expected that future studies will continue to improve this experimental system.

**Fig. 4.**
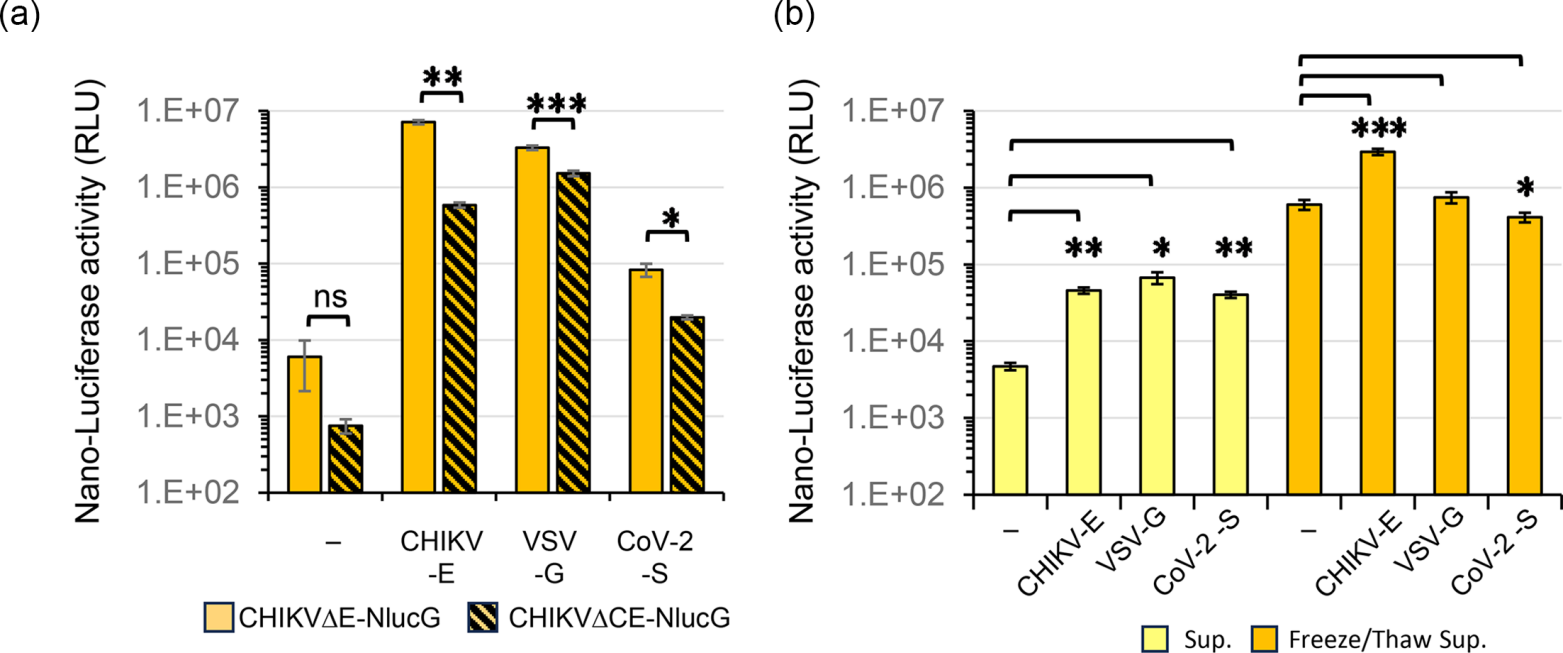
Cell-free NLuc molecules were released into the supernatant. (**a**) Detection of NLuc activity expressed in BHK/hACE2 cells infected with pCHIKVΔE (or ΔCE)-Nluc-GFPbsr/empty, CHIKV-E, VSV-G or CoV-2-S. The NLuc activity of the cell lysate equivalent to 1 in 500 of the volume of cell lysate in 1 well of a 96-well multiplate was measured. (**b**) Detection of NLuc contained in the CHIKVΔE-NlucG/viral envelope protein pseudotype samples obtained as Sup and F/T sup. The NLuc activity of pseudotype samples was detected by using 0.2 µl equivalent of each sample. Data shown are representative of three independent experiments and are expressed as the mean±sd. Statistical significance was evaluated by an unpaired two-tailed *t*-test. *Significant at *P*<0.05; **significant at *P*<0.01; ***significant at *P*<0.001.

Next, according to the manufacturer’s protocol, we tried to detect the infectivity of these pseudotypes by measuring NLuc activity using the Nano-Glo^®^ Luciferase Assay System (Promega Corporation, Madison, WI). However, the background values were generally high and sometimes deviated from the number of GFP-positive cells detected (Fig. 4a). The deviation may have been due to the existence of free NLuc, free NLuc still fusing capsid protein or NLuc packaged in some kinds of particles that were released into the supernatant (Fig. 4b).

We therefore constructed firefly luciferase (luc)-expressing pseudotypes using the pCHIKVΔE-luc vector plasmid and viral envelope expression vector plasmids in the same manner as CHIKVΔE-NlucG/viral envelope protein pseudotypes (Fig. S5). The backgrounds of firefly luciferase activity of the CHIKVΔE-luc/-inoculated cells were lower than that of NLuc, and the infectivities of these CHIKVΔE-luc/viral envelope protein pseudotypes were similar to the FFU of CHIKVΔE-NlucG/viral envelope protein pseudotypes (Figs 2 and S3).

**Fig. 5.**
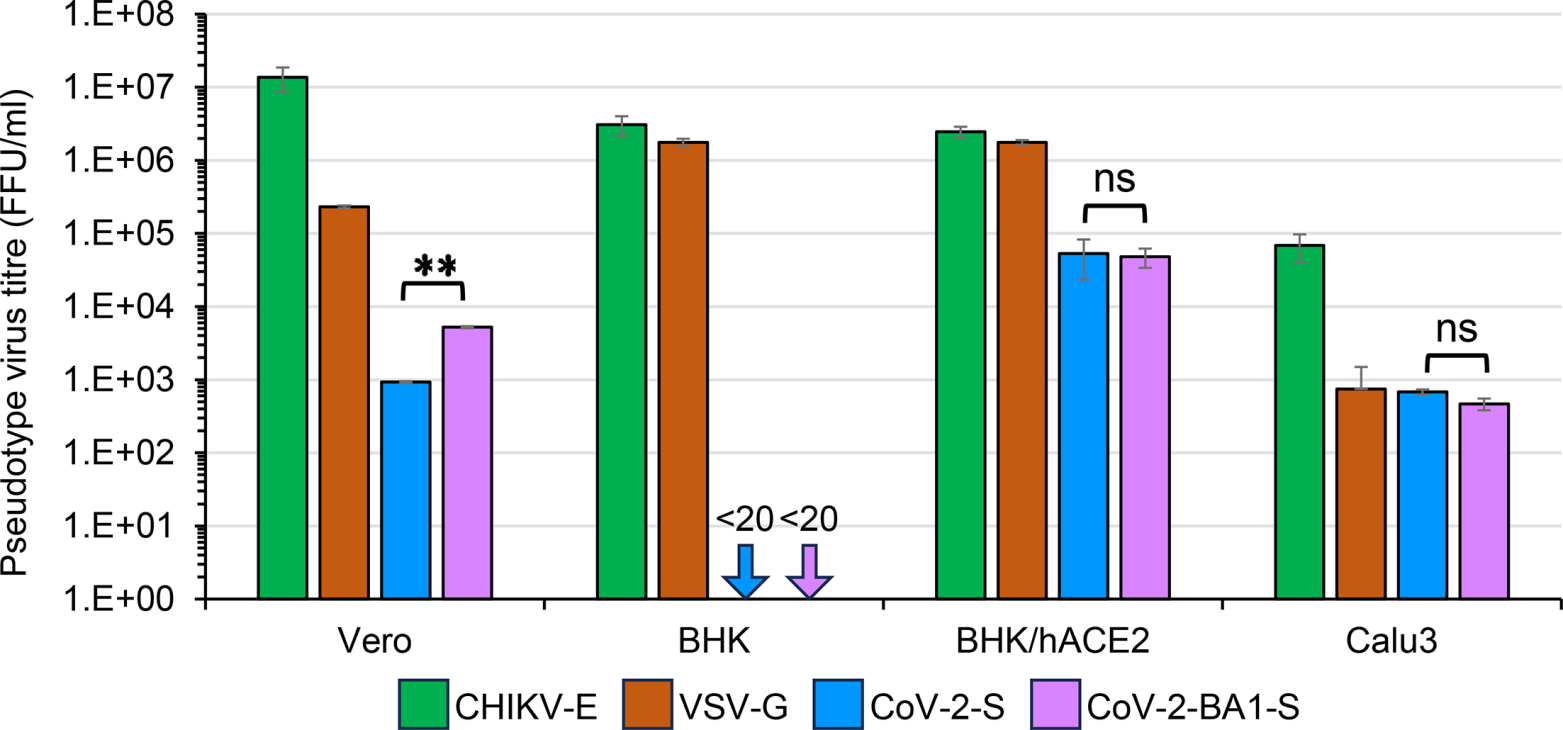
Susceptibilities of cultured cell lines to CHIKVΔE-NlucG/viral envelope protein pseudotypes. Vero, BHK, BHK-hACE2 and Calu3 cells that had been plated 2 days earlier were inoculated with serially diluted pseudotype viruses, and the titre of pseudotype viruses was determined by counting the GFP foci detected by fluorescence microscopy at 1 day after the inoculation. The pseudovirus titres (FFU per millilitre) shown are representative of three independent experiments performed in duplicate or triplicate and are expressed as the mean±sd. Statistical significance was evaluated by an unpaired two-tailed *t*-test. **Significant at *P*<0.01; ns: not significant.

In addition to BHK and BHK/hACE2 cells, pseudotyped infectivity was examined in Vero and Calu3 cells, which express ACE2 and are susceptible to SARS-CoV-2. Vero cells were more susceptible to infection by native-form pseudotype, CHIKVΔE-NlucG/CHIKV-E, than BHK and BHK/hACE2 cells, while the CHIKVΔE-NlucG/VSV-G infectivity was higher in BHK and BHK/hACE2 cells than in Vero cells (Fig. 5). These results indicate that the higher susceptibility of BHK/hACE2 cells of CHIKVΔE-NlucG/CoV-2-S (or CoV-2-BA1-S) compared to Vero cells was not responsible for the capacity for CHIKV gene expression but was responsible for the results of different infectivity via envelope proteins bearing pseudotypes (Fig. 5). BHK/hACE2 cells were more susceptible to infection of CHIKVΔE-NlucG/CoV-2-S than Vero cells, which was similarly observed in native SARS-CoV-2 infection (Fig. S2), that also support these results. In addition, a difference in infectivity was observed between CHIKVΔE-NlucG/CoV-2-S and CHIKVΔE-NlucG/CoV-2-BA1-S in Vero cells but not in BHK/hACE2 or Calu3 cells, suggesting that the spike protein of SARS-CoV-2 Omicron BA.1 showed slightly higher binding affinity with African green monkey ACE2 than the spike protein of the prototype Wuhan strain SARS-CoV-2. It has been reported that the Omicron RBD (strain BA.1) binds to ACE2 more strongly than does the prototypic RBD from the original Wuhan strain [58], and its binding affinity of hACE2 and RBD increased in the order of wild type (Wuhan strain)<Beta<Alpha<Gamma< BA.1 [59,60]. Recently, Tachibana *et al*. reported that the SARS-CoV-2 Omicron BA.1 showed a significantly lower binding affinity with human ACE2 than the prototype SARS-CoV-2 [61]. When pseudotype titres were adjusted for the titres in Vero cells, the relative infectivity of CHIKVΔE-NlucG/CoV-2-S to the human ACE2-expressing cells was higher than that of CHIKVΔE-NlucG/CoV-2-BA1-S, and the binding affinity of CoV-2-S to hACE2 appeared to be higher than that of CoV-2-BA1-S. Unfortunately, however, the absolute binding affinity of actual virus particles could not be determined in our system. Either way, the difference in binding affinity for hACE2 between CoV-2-S and CoV-2-BA1-S does not appear large enough to affect WT virus growth.

Taken together, these results indicate that the infection of the pseudoviruses produced by our pseudotype virus system strictly depends on the expression of viral membrane proteins and viral receptors in target cells, there is no nonspecific infection and the infectious titre is sufficient for virological studies. Our established pseudotype virus system can be used as a robust platform to study various virus infections and for screening and in-depth evaluation of neutralizing antibodies and antiviral agents.

Although we successfully established a novel pseudotyped virus system using recombinant alphavirus genomes, a fundamental question remains unresolved: how is the core incorporated into CHIKVΔE-NlucG/(VSV-G or CoV-2-S) virions, and how does rCHIKV-gRNA become encapsidated in CHIKVΔCE-NlucG/(VSV-G or CoV-2-S) virions? It is well known that the interaction between the viral core and the plasma membrane, which is essential for budding, relies on the hydrophobic residues within the cytoplasmic domain of E2. This interaction is considered highly specific. Therefore, it is puzzling how the core could be packaged into CHIKVΔE-NlucG/(VSV-G or CoV-2-S) particles in the absence of the native envelope proteins. Similarly, how rCHIKV-gRNA is incorporated into CHIKVΔCE-NlucG/(VSV-G or CoV-2-S) virions remains unclear. One possibility we considered is that the large quantity of synthesized rCHIKV-gRNA may stochastically enter vesicles with sufficient frequency. However, this remains a speculative hypothesis, as we could not provide direct evidence. Further investigation is needed to elucidate these mechanisms.

Finally, a logical consequence of our findings in this study is that an alphavirus-derived replicon RNA vaccine, such as the one reported by Erasmus *et al*. [62], could have replicating competent virus activity with cell-to-cell transmission. Replicon RNA vaccines have the potential to become a novel virus species whose host cell range is ACE2 receptor-expressing cells. Therefore, we should exercise due caution in using replicon-type vaccine constructs expressing membrane proteins.

## Supplementary material

10.1099/jgv.0.002138 Uncited Supplementary Material 1.

## Abbreviations

ACE2: angiotensin‑converting enzyme 2
arbovirus: arthropod-borne virus
BHK: baby hamster kidney fibroblast cell line
BHK cell: baby hamster kidney fibroblast cell line
BHK/hACE2: BHK cells stably expressing human ACE2
C: capsid gene (structural protein)
CHIKV: chikungunya virus
CMVenh: cytomegalovirus early enhancer sequence
CMVp/CAGp: CMV early enhancer/chicken β‑actin promoter in pCAGGS
CoV-2-S: coronavirus 2 spike protein
DMEM: Dulbecco’s Modified Eagle Medium
E: envelope gene (includes E3, E2, 6 K, and E1 proteins of CHIKV)
FBS: fetal bovine serum
FFU: focus-forming units
GFPbsr: CpG‑free GFP:Bsr fusion gene
hACE2: human angiotensin-converting enzyme 2
hACE2: human ACE2
HDV: hepatitis D virus (ribozyme)
luc: firefly luciferase gene
MLVp: murine leukemia virus promoter sequence
Nluc: NanoLuc luciferase gene
NLucGFPbsr: NanoLuc‑GFP‑Bsr fusion gene cassette
nsP1‑4: Nonstructural proteins 1 to 4 (CHIKV)
RBD: receptor-binding domain
RBD: receptor-binding domain
Rbz: ribozyme sequence (from HDV)
rCHIKV‑gRNA: recombinant CHIKV genome RNA (defective) corresponding to pseudotype backbone
T2A: thosea asigna virus 2A peptide sequence
VSV: vesicular stomatitis virus
VSV-G: VSV glycoprotein
